# Pheochromocytoma: an updated scoping review from clinical presentation to management and treatment

**DOI:** 10.3389/fendo.2024.1433582

**Published:** 2024-12-13

**Authors:** J. S. Saavedra T., Humberto Alejandro Nati-Castillo, L. A. Valderrama Cometa, Wilfredo A. Rivera-Martínez, Josué Asprilla, C. M. Castaño-Giraldo, Leonardo Sánchez S., Mishell Heredia-Espín, Marlon Arias-Intriago, Juan S. Izquierdo-Condoy

**Affiliations:** ^1^ Family Medicine Department, Universidad Javeriana, Cali, Colombia; ^2^ Interinstitutional Group on Internal Medicine (GIMI 1), Department of Internal Medicine, Universidad Libre, Cali, Colombia; ^3^ Organ and Tissue Transplant Unit, Clínica Imbanaco, Cali, Colombia; ^4^ Facultad de Medicina, Universidad de Antioquia, Medellin, Colombia; ^5^ Division of Pathology, Clínica Imbanaco, Grupo Quirónsalud, Cali, Colombia; ^6^ Facultad de ciencias de la Salud, Universidad del Quindío, Armenia, Colombia; ^7^ One Health Research Group, Universidad de las Americas, Quito, Ecuador

**Keywords:** pheochromocytoma, paraganglioma, hormonal imbalance, diagnosis, management, treatment

## Abstract

Pheochromocytomas and paragangliomas (PPGLs) are rare neuroendocrine tumors derived from chromaffin cells, with 80–85% originating in the adrenal medulla and 15–20% from extra-adrenal chromaffin tissues (paragangliomas). Approximately 30–40% of PPGLs have a hereditary component, making them one of the most genetically predisposed tumor types. Recent advances in genetic research have classified PPGLs into three molecular clusters: pseudohypoxia-related, kinase-signaling, and *WNT*-signaling pathway variants. Specifically, the detection of *SDHB*-related tumors indicates an increased risk of metastatic disease, which may impact decisions regarding functional imaging in patients with high suspicion of metastasis and influence targeted treatment strategies. Diagnosis of PPGLs primarily relies on biochemical testing, measuring catecholamines or their metabolites in plasma or urine. However, molecular testing, functional imaging, and targeted therapies have greatly enhanced diagnostic precision and management. Personalized treatment approaches based on genetic profiling are becoming integral to the clinical management of these tumors. In South American countries like Colombia, functional imaging techniques such as positron emission tomography/computed tomography (PET/CT) with tracers like 18F-DOPA, 18F-fluorodeoxyglucose (18F-FDG), and 68Ga-DOTA-conjugated somatostatin receptor-targeting peptides (68Ga-DOTA-SST) are used to guide follow-up and treatment strategies. Radionuclide therapy with lutetium-177 DOTATATE is employed for patients showing uptake in 68Ga-DOTA-SST PET/CT scans, while access to 131-MIBG therapy remains limited due to high costs and availability. Recent clinical trials have shown promise for systemic therapies such as sunitinib and cabozantinib, offering potential new options for patients with slow or moderate progression of PPGLs. These advancements underscore the potential of personalized and targeted therapies to improve outcomes in this challenging patient population.

## 1 Introduction

Pheochromocytomas and paragangliomas (PPGLs) are closely related tumors that originate from neuroendocrine cells, arising from chromaffin cells in the adrenal medulla and neural crest progenitors located outside of adrenal gland, respectively (1). These tumors are characterized by a proliferation of chromaffin cells arranged in clustered or trabecular patterns (2, 3), Although rare, occurring in fewer than 0.1% of individuals per million (4, 5), pheochromocytomas and sympathetic paragangliomas in particular require prompt treatment to reduce associated morbidity and mortality (3, 6).

Paragangliomas (PGLs) arise in sympathetic and parasympathetic paraganglia (7–9). Those in the head and neck are predominantly parasympathetic, typically non-metastatic, and often present as palpable masses, while abdominal PGLs arise from the sympathetic neuroendocrine system and share origins with pheochromocytomas (2). Notably, carotid body tumors, a type of PGL, are highly vascularized glomus tumors located at the carotid bifurcation, where the external and internal carotid arteries diverge (10–13).

PPGL, whether located in the adrenal medulla or at extramedullary sites, secrete excessive amounts of catecholamines, adrenaline, noradrenaline, and/or dopamine. There is a subdivision based on the genotype of PPGLs in cluster 1 for variants in pseudohypoxia genes, cluster 2 for alterations in the kinase pathway and cluster 3 in WNT signaling. This classification in cases such as *SDHB*-related tumors defines the prognosis of developing metastatic disease and can modify the conduct of treatment and surveillance (14–17). Cluster 1 tumors such as *VHL* typically produce norepinephrine, whereas cluster 2, *MEN2*, or *NF1* tumors are more likely to produce epinephrine, and *SDHB, SDHC*, and *SDHD*-related tumors may secrete dopamine and norepinephrine (18, 19).

## 2 Epidemiology

Pheochromocytomas occur with an estimated incidence of 0.05%, primarily in adults aged 30-50 (20). They constitute around 4% of incidental adrenal masses and are implicated in approximately 0.1% of hypertension cases. Demographically, pheochromocytoma affects adults of both sexes, typically between 30 and 50 years of age, presenting, and they appear with similar frequency in both adrenal glands (2, 4, 5).

Around 20% of PPGL present metastases. 70% of pheochromocytomas are sporadic, while the remaining 30% are associated with hereditary syndromes such as multiple endocrine neoplasia type 2 (MEN II), von Hippel-Lindau disease (VHL), neurofibromatosis type 1 (NF1), and familial paraganglioma syndrome (21, 22).

Traditionally, pheochromocytomas were managed under the “10% rule,” (23), which suggested that 10% occur in children, 10% are extra-adrenal, 10% are familial, 10% are bilateral in adrenal glands, and 10% are metastatic (24). However, it is currently not recommended to refer to it as “the 10 percent tumor,” since approximately 25% of patients with apparently sporadic pheochromocytoma may carry pathogenic variants (18).

## 3 Clinical predictor of metastasis

All PPGLs have metastatic potential, however histological characteristics do not allow differentiating “benign” from “malignant” tumors, so the latest WHO update recommends changing these terms to metastatic, when there is evidence of distant tumor (25). The ESMO guidelines, for their part, suggest using the definition of “high risk of metastasis” when one or more of the following criteria are present: (a) tumor size greater than or equal to 5 cm; (b) any extra-adrenal PPGL; (c) known germline *SDHB* pathogenic variant; or (d) plasma 3MT > 3 times above the upper limit of normal (26). Certain tumors, particularly *SDHB*-related PGLs of the head and neck, can produce dopamine. In such cases, detecting its metabolite, methoxytyramine, in blood has shown only a modest improvement in detecting head and neck PPGL (27, 28). Although its use as a metastatic risk marker has been considered, its performance remains limited (29), and it is unavailable in most countries around the world. Management guidelines do not recommend its measurement (30, 31).

**Figure 1:**
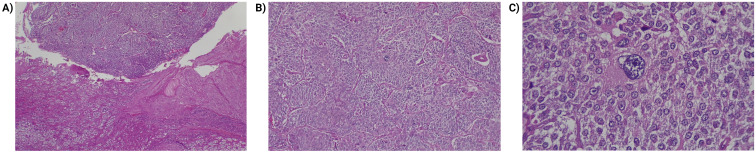
Histopathologic findings of pheochromocytoma. **(A)** Transition between the usual histology adrenal cortex (lower part of the image) and the tumor (upper part). Hematoxylin and eosin 40x. **(B)** Pheochromocytoma, cells arranged in a pattern in nests (zellballen) and trabeculae. Hematoxylin and eosin 40x. **(C)** Pheochromocytoma, lesion cells are large, polygonal, show fine, granular cytoplasm. Note the presence of pleomorphism. Hematoxylin and eosin 400x.

Histologically, pheochromocytomas are characterized by pleomorphic cellular nests, with large ball-shaped chromaffins that show strong positivity for chromogranin A, synaptophysin, CD56, and focal S100 (**Figure 1**). There are no definitive histological criteria for malignancy; thus, the term “metastatic tumor” is preferred when chromaffin tissue invasion is confirmed beyond the site of origin into distant organs (32, 33). It is also important to assess histological criteria for aggressive biological behavior, including an insular pattern of growth, mitotic activity, and invasion of capsular blood and lymphatic vessels (34). Scores like PASS (Pheochromocytoma of the Adrenal Gland Scaled Score), Grading of Adrenal Pheochromocytoma and Paraganglioma, COOPS (Composite Pheochromocytoma/Paraganglioma Prognostic Score) and multivariate predictive models (SGAP-Score and ASES/ASS-Score) have been developed to identify PPGL patients with increased metastatic potential. However, immunohistochemistry may yield inconsistent results, lacks molecular testing which is more reliable, and scores such as PASS lack clinical validation studies supporting their application (35). These methods are not currently utilized in Colombia or Ecuador (30, 36).

On the other hand, there are more promising clinical predictors, TNM staging may be correlated with overall survival in PPGL. Jimenez et al. (37), found that a large primary tumor, an extra-adrenal location, infiltration of surrounding tissues by the primary tumor, and regional lymph node metastasis are associated with a higher risk of distant metastasis and consequently decreased overall survival, and in turn, patients with distant metastasis (stage IV) have the worst prognosis.

## 4 Hereditary and phenotypic pattern

### 4.1 Susceptibility genes

PPGL are associated with germline pathogenic variants at higher rates than any other solid tumor. Rates of germline pathogenic variants vary by tumor type: 25% in pheochromocytoma, 40% in PGL, and up to 50% in patients presenting with metastatic disease. Patients with pathogenic variants generally present with PPGL at a younger age and are more likely to have multifocal disease (18). 30% to 40% of cases occur in the context of a genetic syndrome, however, in almost half of apparently sporadic PPGLs somatic pathogenic variants are found in one of the susceptibility genes, which means that at least three quarters can be classified into a defined cluster.

The susceptibility to pheochromocytoma can be linked to germline pathogenic variants in the *RET* proto-oncogene and tumor suppressor genes such as *von Hippel-Lindau* (*VHL*), and *Neurofibromatosis type 1* (*NF1*) (2, 38, 39). Hereditary paraganglioma syndromes are caused by pathogenic variants in the succinate dehydrogenase subunit (SDHx) genes: *SDHD, SDHAF2, SDHC, SDHB, SDHA* (Paraganglioma syndromes 1-5, respectively) (40). The *VHL* gene, located on the short arm of chromosome 3 (3p25.3), has over 1,500 identified pathogenic variants in patients with VHL disease, with 20% of these being *de novo* pathogenic variants, as observed in both PGLs and pheochromocytomas (8, 23, 41). Other pathogenic variants in susceptibility genes such as *TMEM127, MAX, FH* and *MDH2* are associated with PPGL syndromes with a lower pathogenic variant frequency (42, 43).

The discovery of syndromes linked to pathogenic variants in genes has shown that the probability of metastasis may vary, being no higher than 12% in *SDHC, THEM127, NF1, VHL, RET* and *MAX*, but for *SDHD, SDHA* and *SDHB* it reaches 29, 66 and 75%, respectively (30, 42, 44–46).

### 4.2 Inheritance pattern

Pheochromocytoma is currently known to result from disorders with an autosomal dominant inheritance pattern in most cases, such as multiple endocrine neoplasia type 2 (*MEN-2*) (presenting manifestation is medullary thyroid cancer in 60%, medullary thyroid carcinoma and synchronous pheochromocytoma in 34%, and pheochromocytoma in 6%. 72% have bilateral pheochromocytoma, 82% of tumors are synchronous and are unlikely to be metastatic) and von Hippel-Lindau disease (with retinal angioma, central nervous system hemangioblastoma, renal cell carcinoma, pancreatic cysts, and epididymal cystadenoma) (24, 43, 47). The precise frequency of these syndromes in patients with pheochromocytoma is not fully known (2, 38, 39, 43). The exception are pathogenic variants in the *SDHAF2* and *SDHD* genes, in which maternal imprinting occurs with silencing of the maternal allele and therefore only pathogenic variants inherited from the father will cause the disease, they probably have the highest penetrance, greater than 50% and are usually associated with PPGL in the head, neck and chest (30, 44–46). On the other hand, the inheritance pattern for variants in the *MAX* gene is not clear; it is believed that its penetrance is high and it usually presents as bilateral pheochromocytomas and abdominal PGL (48).

### 4.3 Molecular phenotype

**Figure 2:**
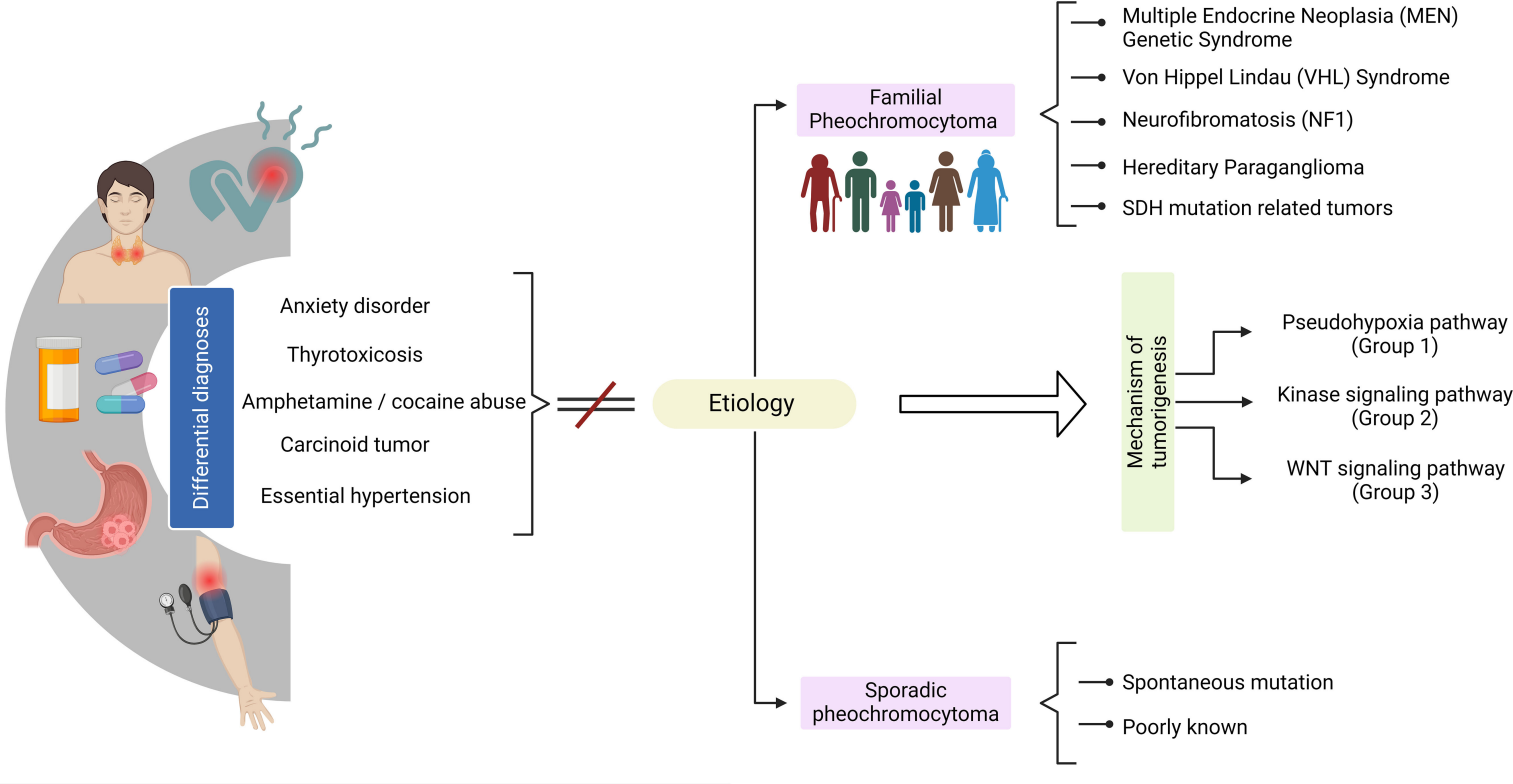
Clinical overview of pheochromocytoma diagnosis.

The genome of PPGL has been characterized, providing valuable insights into the genetic factors driving tumorigenesis and the degree of genetic instability (49, 50) (**Figure 2**). These tumors are generally characterized by a relatively low degree of genetic instability at both the nucleotide and chromosome levels. Although there are rare tumors that behave differently from others, most of the genes associated with the development of PPGL are categorized into three clusters based on the mechanism of tumorigenesis (49–51):

- Pseudohypoxia pathway (cluster 1), tumors that infiltrate stromal cells, which have been associated with pathogenic variants in the genes *EGLN1, EGLN2, DLST, FH, IDH3B, MDH2, SDHA, SDHAF2, SDHB, SDHC, SDHD, VHL, EPAS1, IDH1*, and *IDH2*.
- Signaling kinase pathway (cluster 2) has been associated with pathogenic variants in the *NF1, MAX, MERTK, MET, MYCN, RET*, or *TMEM127* genes.
- In cluster 3, *MAML3* fusion gene and *CSDE1* somatic pathogenic variants affect and overactivate the Wnt/β-catenin pathway, which is responsible for the regulation of metabolism, angiogenesis, proliferation, and invasion.

Given the above pathogenic variants, one of them stands out for its potential usefulness in the prognosis of the disease. SDH (succinate dehydrogenase) is an important enzyme in energy formation pathways, taking action in the Krebs cycle and in the Electron Transfer chain within the mitochondria, where is conforming the complex ll with four functional subunits: A, B, C, and D. Inactivating germline pathogenic variants results in loss of function of SDH and, therefore, an elevation in succinate levels which diffuses back to the cytoplasm and inhibits prolyl hydroxylases, resulting in further stabilization of the Hypoxia-Inducible Factor (HIF) pathway of tumorigenesis. *SDHB* related tumors, are commonly found in the abdomen, have a high potential for recurrence, local and distant metastasis compared to *SDHD* and *SDHC* tumors, which are commonly found in the head and neck areas, each of them requiring follow-up for the possibility of relapse or extension of the disease (52, 53).

## 5 Clinical presentation

**Figure 3:**
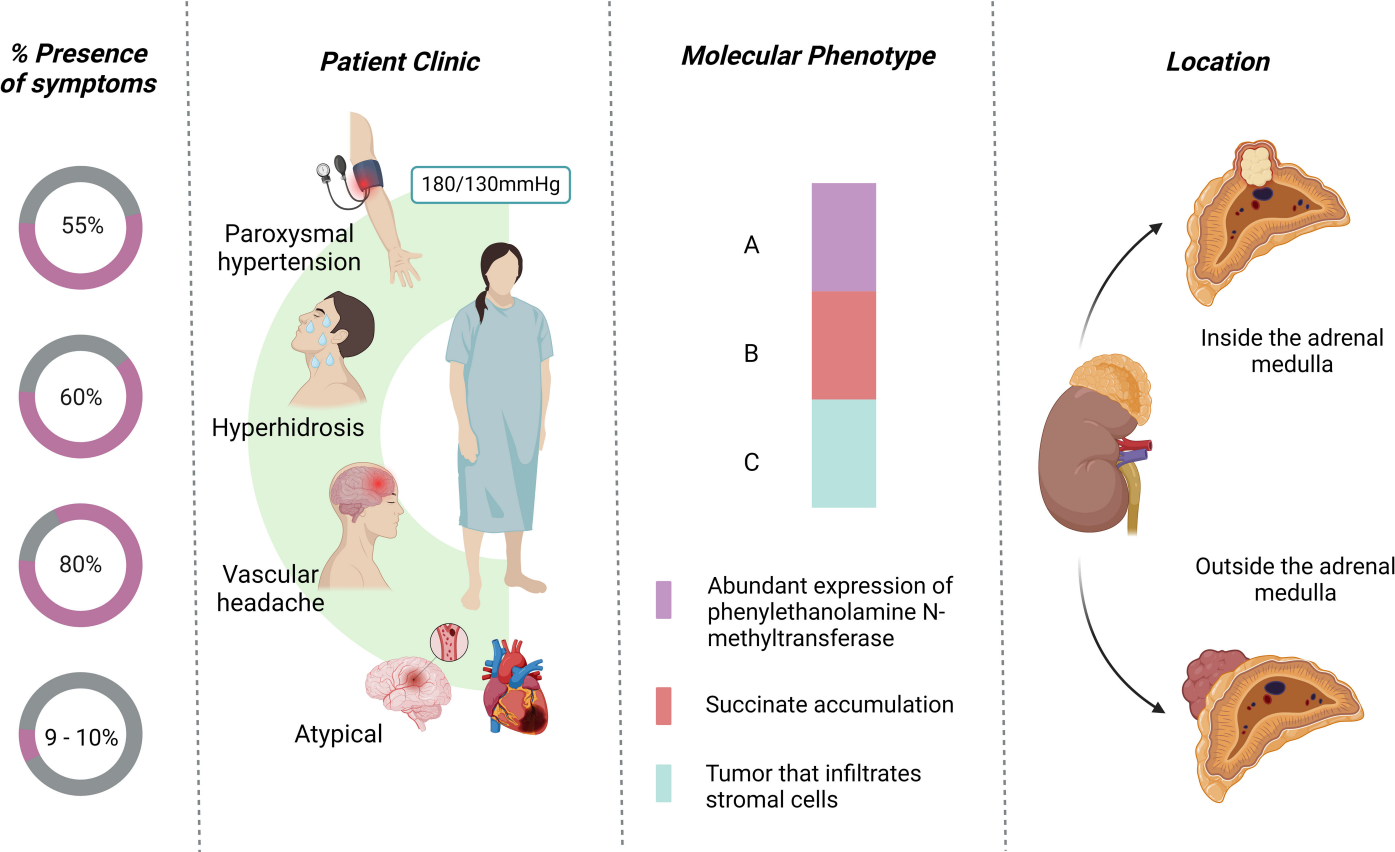
Characteristics of the presentation of pheochromocytoma.

Patients with pheochromocytoma are often described as presenting with the “classic triad” of diaphoresis, headache, and palpitations, typically accompanied by hypertension (22). In a review of 200 cases, Ando et al. found that PPGL attacks are associated with multisystem involvement, 99% cardiac damage, 44% pulmonary damage, and 21.5% renal damage (54). Sustained hypertension is observed in approximately 50-55% of cases, while paroxysmal hypertension occurs in 30-45% (24, 48, 55). Additionally, hyperhidrosis occurs in 60% of cases, often accompanied by hypertensive crises (56, 57). Headache is one of the most common symptoms, and may occur in up to 40% of cases (4, 54, 58). Headache is related to the transient elevation of blood pressure. Instead of sustained hypertension, PPGL headache can occur suddenly as cluster headache, however they are usually bilateral, associated with hypertension and hyperhidrosis (**Figure 3**) (54, 59, 60).

PPGL is presented with sustained or episodic hypertension, sweating, palpitations, hyperglycemia, and glycosuria (5). Although some tumors produce dopamine, the majority secrete noradrenaline and adrenaline (5, 61). PPGLs may experience episodes of severe hypertensive crisis, with an increased likelihood of developing acute kidney injury. Over time, the vasoconstrictive effect of catecholamines released by the tumor leads to chronic kidney disease being a potential complication in PPGLs (62).

Giant pheochromocytomas, defined as those larger than 7 cm, are rare (63–65). These tumors often do not present with classic symptoms; most patients report vague discomfort, and a few may present with a palpable abdominal mass (34, 65, 66).

**Table 1** summarizes the clinical translation of the conditions in patients with pheochromocytoma and sympathetic paragangliomas, and their possible complications.

**Table 1:** Synthesis of clinical expression from diagnosis to complications of pheochromocytoma and sympathetic paragangliomas.

Physiologic manifestations of catecholamines excess	
Compromised tissue	Effect
Heart	Tachycardia
	Tachyarrhythmias
	Increase myocardial oxygen consumption
	Myocarditis
	Cardiomyopathies
	Arterial hypertension
Blood vessels	Plasmatic volume decrease
Intestine	Intestinal relaxation, altered intestine motility
Pancreas	Carbohydrate intolerance due to Beta cells regulation changes and insulin release suppression.
	Hyperglycemia
	Glucosuria
Adipose tissue	Lipolysis due to free fatty acids increase
Apocrine glands	Diaphoresis due to stimulation
Clinical presentation of pheochromocytoma	
Symptoms	% of presence
Dizziness	67%
Headache	59%
Palpitations	50%
Diaphoresis	50%
Weight loss	30%
Syncope episodes	40%
Anxiety	19%
Signs	% of presence
Sustained hypertension	48%
Paroxysmic hypertension	44%
Hypertension	92%
Tachycardia	15%
Orthostatic hypotension	12%
Pheochromocytoma complications	
Affected system	Clinical manifestation
Cardiovascular	Arrythmia, ventricular tachycardia, Torsade de Pointes, ventricular fibrillation.
Respiratory	No cardiogenic pulmonary edema
Gastrointestinal	Ileo, constipation.
Renal	Renal infarction, renal artery stenosis.
Metabolic	Physiological Manifestations of Catecholamine Excess

Signs and symptoms alone are often nonspecific, and relying solely on them can lead to diagnostic errors. To improve diagnostic accuracy, efforts have been made to develop diagnostic scores that emphasize the most specific clinical features, such as pallor, hyperhidrosis, and palpitations (**Table 2**) (67).

**Table 2:** Scoring system to classify the probability of pheochromocytoma/paraganglioma according to symptoms and clinical signs.

Symptoms and signs	Score per item (total score from -1 to +7)*
Pallor	+ 1 point
Hyperhidrosis	+ 1 point
Palpitations	+ 1 point
Tremor	+ 1 point
Nausea	+ 1 point
Heart rate of 85 bpm or higher	+ 1 point
Body mass index (BMI) < 25 kg/m2	+ 1 point
Obesity (BMI > 30 kg/m2)	- 1 point
Odds by group	
Group 1 Related Pheochromocytoma	More likely to be associated with lower scores and sustained hypertension
Group 2-related pheochromocytoma	More likely to be associated with higher scores
	Episodic presentation of symptoms, including tremor, anxiety/panic, and pallor, and older age at first diagnosis

*Total score 3 or more indicates a 5.8-fold increased probability of pheochromocytoma/paraganglioma.

## 6 Diagnosis

### 6.1 Laboratory tests

Diagnostic testing is crucial for confirming PPGL, although it is typically performed under specific conditions, such as the presence of known germline pathogenic variants, a history of PPGL, detection of an incidental adrenal or extra-adrenal mass suggestive of these tumors, or presentation with relevant clinical signs and symptoms (17). The 24-hour plasma metanephrine test offers the highest sensitivity (96%) and a specificity of 85% (68, 69). Studies have shown that plasma normetanephrine levels above 2.5 pmol/mL or metanephrine levels above 1.4 pmol/mL are highly indicative of pheochromocytoma, with 100% specificity. To reduce false-positive results, it is recommended that blood samples be collected with the patient in a supine position after being recumbent for at least 30 minutes (2).

The 24-hour urinary collection for catecholamines and metanephrines is another accessible test, providing a sensitivity of 87.5% and a specificity of 99.7%. Linking urinary metanephrine levels to urinary creatinine further enhances accuracy (70).

It is important to note, however, that catecholamine measurements are only informative when levels are elevated, as in a catecholaminergic crisis, which is typically not present at diagnosis. Physical activity and psychological stress can increase plasma and urinary metanephrine levels; thus, minimizing these factors before sampling is recommended. Additionally, various commonly used medications—including tricyclic antidepressants, monoamine oxidase inhibitors, atypical antipsychotics, selective adrenergic receptor blockers, stimulants, sympathomimetics, paracetamol, sulfasalazine, and amoxicillin—can interfere with test results, so these should be avoided prior to testing, if possible (71).

The clonidine suppression test helps differentiate elevated plasma norepinephrine levels due to sympathetic nerve release from those due to pheochromocytoma (70, 72). Clonidine, a centrally acting alpha-2 agonist, suppresses neuronal norepinephrine release (73, 74). However, chromaffin cells in pheochromocytomas are not regulated by clonidine and continue to release catecholamines inappropriately (73, 75). This test is thus useful in evaluating false-positive results for pheochromocytoma (76), and to reliably differentiate pheochromocytomas from essential hypertension. This test has a sensitivity of 97% and a specificity of 100%. A decrease in plasma norepinephrine levels below 50% following clonidine administration is considered normal, while persistent elevations suggest pheochromocytoma (73, 74).

### 6.2 Imaging study

Once biochemical analyses suggest the presence of pheochromocytoma, imaging studies are recommended to locate the tumor. A computed tomography (CT) scan of the abdomen and pelvis is typically the initial imaging test of choice (2). CT has a sensitivity of 88%, being useful in the localization of pheochromocytomas larger than 1.3 cm in diameter with an accuracy of 90 to 95%, and represents the most common imaging method used in the diagnosis of pheochromocytomas (77). In comparison, magnetic resonance imaging, with differences in access and costs, offers better spatial resolution (65). Another imaging modality, 123I-metaiodobenzylguanidine (MIBG) scintigraphy, is especially effective for detecting adrenal and extra-adrenal pheochromocytomas (78). Magnetic resonance imaging in the diagnosis shows a differentiated appearance with a sensitivity of 100%, as well as Scintigraphy131 -MBG (sensitivity of 100%), this analogue is located in the adrenergic tissue, it is especially useful to locate extra-adrenal pheochromocytomas (78, 79).

In functional imaging, ^68^Ga-DOTA-conjugated somatostatin receptor-targeting peptide (^68^Ga-DOTA-SST) positron emission tomography (PET/CT) has a detection rate of 93% (95% IC 91-95%) (80), making it the first line of functional imaging for patients without known germline pathogenic variants. However, due to the limited availability of 68Ga-DOTA-SST in Latin America, 18F-fluorodeoxyglucose (18F-FDG) PET/CT is often used as a second imaging option in these populations, with a detection rate of 74% (95% CI, 46-91%). 18F-L3,4-dihydroxiphenylalanine (18F-DOPA) PET/CT is available in Colombia and is the first choice in Hereditary pheochromocytoma cluster 2, with an 80% detection rate (95% CI, 69-88%) (17). Alternatively, a vena cava sample can be used to determine plasma catecholamines and metanephrines (81).

### 6.3 Genetic testing

Since 35-45% of PPGL patients may harbor pathogenic germline variants, genetic testing is recommended for all diagnosed cases, regardless of patient or family history (42, 44, 68). Bilateral tumors and early-onset cases are often associated with inherited syndromes such as VHL, MEN2, and NF1. At a minimum, testing should include *FH, NF1, RET, SDHB, SDHD*, and *VHL* genes. Testing for *MEN1, SDHA, SDHAF2, SDHC, TMEM127*, and *MAX* is also advised. Carrier testing should be offered to asymptomatic first-degree relatives (and to second-degree relatives in the case of *SDHD* and *SDHAF2*, which exhibit maternal imprinting) (42, 44). Following identification of a pathogenic variant, first-degree relatives should be screened (82).

### 6.4 Immunohistochemistry

Pathogenic variants in *SDHB* in PPGL patients are associated with a higher risk of tumor progression, and several studies have shown that *SDHB* pathogenic variation can be detected by the loss of *SDHB* staining in immunohistochemistry (IHC). This staining loss can serve as an independent IHC biomarker for prognosis. However, this approach is not universally applicable; under normal conditions, SDHB functions as part of the succinate dehydrogenase complex. Pathogenic variants in any gene encoding other complex subunits or auxiliary factors (such as *SDHD, SDHC, SDHA*, or *SDHAF2*) disrupt the assembly and functionality of the entire complex, resulting in an absence of *SDHB* staining on IHC (83).

## 7 Management and treatment

### 7.1 Surgical resection of the tumor

In most cases, resection of pheochromocytomas smaller than 5 cm can be effectively performed using minimally invasive laparoscopic surgery. This approach offers significant advantages, including reduced blood loss, less pain, precise dissection, shorter hospital stays, and fewer postoperative complications. The transabdominal approach provides a broader field of view and more space for maneuvering, making it suitable for bilateral tumor removal. Conversely, the retroperitoneal approach allows for unilateral resection with shorter distance to the tumor, minimizing the risk of injury to abdominal organs (2, 30, 84, 85).

Evidence suggests that patients with PPGL associated with malignancy predictors—such as primary tumor size over 5 **cm**, extra-adrenal location, or *SDHB* germline pathogenic variants—and those undergoing resection of a primary tumor with synchronous metastases may benefit more from open laparotomy with lymph node dissection (86). In cases with larger tumors or evidence of local invasion, open adrenalectomy may be preferable to ensure complete resection and minimize the risk of capsular rupture, which can lead to tumor seeding, fragmentation, peritoneal dissemination, and local recurrence due to periadrenal invasion (18, 30).

### 7.2 Preoperative stabilization

Preoperative stabilization is crucial to reducing the risk of uncontrolled hypertension, tachycardia, and volume expansion during surgery (11, 24, 59). According to the Endocrine Society, the preferred preoperative preparation involves alpha-adrenergic receptor blockers. A typical regimen includes phenoxybenzamine, starting at 10 mg orally twice daily and carefully increasing to a maximum of 1 mg/kg/day; however, its availability is limited in many countries (87). Alternatively, selective alpha-1 antagonists like doxazosin are commonly used in regions such as Latin America. They reduce the risk of postoperative hypotension but require close monitoring due to the potential for orthostatic hypotension upon initiation. Beta-blockers (e.g., propranolol, atenolol) are added 3–4 days after starting alpha-blockers to control tachycardia (87). Calcium channel blockers, such as amlodipine or nifedipine, can also be used as additional agents to manage hypertension. Increased water and salt intake is recommended 10–14 days before surgery to prevent postoperative hypotension (2, 20, 81).

During perioperative management, surgeons should minimize tumor manipulation to prevent catecholamine surges and avoid tumor spillage, especially in cystic lesions. Early control of the adrenal vein is also recommended to manage the sudden decrease in peripheral vascular resistance following tumor removal (88). Preoperative biopsies are generally not recommended (89).

### 7.3 Surveillance and restaging of patients with metastatic PPGL

For patients with secretory metastatic PPGL, biochemical monitoring of 24-hour urinary fractionated metanephrines or free plasma is recommended at least every six months, as large increases may indicate disease progression. In nonsecretory metastatic PPGL, further measurements of plasma or 24-hour urinary metanephrines are generally unnecessary unless there is a germline pathogenic variant indicating persistent risk or if signs and symptoms of secretory disease appear (30).

Expert guidelines, such as those from NANETS, recommend surveillance imaging for metastatic PPGL with cross-sectional anatomic imaging (CT or MRI) every 3–6 months during the first year, and if disease remains stable, every 6–12 months thereafter. For liver metastases, triple-phase CT or MRI with contrast is recommended. In metastatic PPGL cases on systemic therapies, surveillance imaging with CT or MRI is suggested every 3–6 months. Functional imaging is not typically recommended for patients with primary PPGL before or after surgery; however, it can more accurately detect metastatic disease if strongly suspected (30). In such cases, PET/CT has been studied with various radiotracers, including 18F-DOPA, 18F-FDG, and 68Ga-DOTATATE. These tracers are superior to MIBG scintigraphy, which is only used when 131I-MIBG treatment is being considered. Tracer efficacy in cluster 1 PPGL depends on somatostatin receptor uptake, making 68Ga-DOTATATE highly specific and effective for detecting small tumors, and tumor glucose metabolism, which enhances the effectiveness of 18F-FDG in aggressive, undifferentiated tumors (90). For bone-only metastatic disease, both SSTR PET/CT and FDG may be useful for routine imaging surveillance (30). 18F-DOPA’s effectiveness is based on tissue uptake via amino acid transporters, making it particularly suitable for non-metastatic cluster 2 tumors (90).

### 7.4 Non-surgical and novel therapies

Various local and regional therapies, including debulking surgery, cementoplasty, radiotherapy (including stereotactic and CyberKnife), radiofrequency ablation, cryotherapy, and tumor embolization, can manage symptoms associated with catecholamine production, tumor burden, or bone involvement (91–93). For patients with stable disease, low tumor burden, and oligometastatic disease, active surveillance is indicated (30, 92, 93). For resectable lesions, options include primary tumor surgery, oligometastatic disease surgery, and debulking surgery. When surgery is not feasible, and disease progression is rapid, with a high visceral tumor burden or severe symptoms, systemic chemotherapy is recommended to control disease progression and alleviate symptoms (14, 15, 92–94). Current chemotherapy regimens include cyclophosphamide, doxorubicin, dacarbazine, and vincristine, though no first-line drug has been defined. Approximately 37% of patients respond to chemotherapy, although complete responses are uncommon (95, 96). Temozolamide, an oral alternative to dacarbazine, may be an option for patients with pathogenic variants *SDHB* with methylation of the O(6)-methylguanine-DNA methyltransferase (MGMT) promoter. However, chemotherapy generally reduces tumor size and helps control blood pressure in only one-third of patients with metastatic pheochromocytoma-sympathetic paraganglioma (97, 98).

The only FDA-approved treatment for metastatic PPGL, approved in 2018, is high-specific activity iodine-131 metaiodobenzylguanidine (HSA-I-131-MIBG), targeting neuroendocrine cells with a response rate of 30–40% (35). In a multicenter phase 2 trial, 68 patients with advanced PPGL received at least one dose, with 25% (95% CI, 16%-37%) showing durable reductions in antihypertensive medication. Among evaluable patients, 92% achieved either partial response or stable disease within 12 months. Elevated serum chromogranin levels (≥1.5 times the baseline upper limit) decreased in 68% of patients (19 of 28), and median overall survival was 36.7 months (95% CI, 29.9-49.1 months). Common side effects included nausea, myelosuppression, and fatigue, with no hypertensive events (99). A real-world study by Al-Ward et al. reported a 38% objective response rate and an 83% disease control rate in 24 patients with metastatic PPGL, with complete response in two cases, 30% metanephrine normalization, and >50% improvement in 46% of cases. Blood pressure normalized in 56%, though seven patients had reversible grade 3–4 myelosuppression, and one experienced fatal pneumonitis (100). Ultratrace iobenguane 131I, a highly specific 131I-MIBG, is no longer available. In Colombia, 131I-MIBG can be imported, though its high cost—greater than tyrosine kinase inhibitors and chemotherapy—limits availability. Additionally, 50% of patients do not show 131I-MIBG uptake, further restricting its use in Latin America (101). Lutetium-177 DOTATATE/TOC has emerged as an alternative management option in patients with advanced PPGL, however its evidence so far comes from retrospective studies (102, 103). In Colombia, Lutetium-177 DOTATATE/TOC is approved for use in neuroendocrine tumors. In a phase II clinical trial conducted by Reyes et al. Lutetium was shown to be safe and effective in a population of 13 patients with inoperable and progressing advanced neuroendocrine tumors, but no patients with PPGL were included (104).

In addition, for patients with slow/moderate progression, not candidates for radionuclide therapy angiogenesis and proliferative signaling inhibitors have been tested as novel treatments, focusing on the interaction between several growth factors including vascular endothelial growth factor [VEGF], platelet-derived growth factors [PDGF] and others, with tyrosine kinase receptors (105). Sunitinib, which inhibits VEGF1, VEGF2, VEGF3, PDGF-alpha, PDGF-beta, c-kit, fms-related tyrosine kinase 3, and RET proto-oncogene receptors, has demonstrated potential in reducing angiogenesis and tumor cell growth. Small studies have shown a disease control rate of 57–83%, with median progression-free survival ranging from 4 to 13 months (106). The FIRSTMAPP study, a phase II randomized placebo-controlled trial, recently reported that Sunitinib achieved the primary endpoint of 12-month progression-free survival in 36% of patients with progressive metastatic PPGL (90% CI, 23–50%), compared to 19% in the placebo group (90% CI, 11–31%). Grade 3 or 4 adverse effects included asthenia, hypertension, and bone or back pain (107). Another drug, Cabozantinib was evaluated in the Natalie Trial, a single-arm phase II trial with 17 patients and a median follow-up of 25 months. The overall response rate was 25.0% (95% CI, 7.3–52.4), with responses observed in 4 out of 16 patients. Grade 3 adverse events included hand-foot syndrome, hypertension, rectal fistula, QT prolongation, and asymptomatic hypomagnesemia. Additionally, two cases of asymptomatic elevations in amylase and lipase were reported (108). On the other hand, Axitinib, a VEGFR2 inhibitor, has also shown promise, particularly in metastatic pheochromocytomas where the pseudohypoxic tumor environment stimulates VEGF synthesis, promoting angiogenesis. Phase II trials of Axitinib reported a partial response in 36% of patients (105).

New therapies for metastatic PPGL under investigation include Belzutifan, a HIF-2 inhibitor used in VHL disease, currently in a phase II trial (NCT04924075) for PPGL (108). Another HIF-2 inhibitor, DFF332, is currently in a phase I/Ib trial (NCT04895748) as monotherapy and in combination with agents like everolimus, spartalizumab, and taminadenant.

Additional investigational drugs include Olaparib, a poly(ADP-ribose) polymerase (PARP) inhibitor involved in DNA repair, being tested in combination with temozolomide (NCT04394858), and Tipifarnib, a farnesyl transferase inhibitor that supports tumor cell survival (NCT04284774).

Finally, Imipridone, a promising agent targeting caseinolytic protease P (ClpP) and acting as a dopamine-like receptor antagonist, has also garnered interest. In a phase II trial, 10 patients received Imipridone at 625 mg weekly; of these, five showed partial responses, and two had stable disease. In a second cohort, where patients received two doses on consecutive days weekly, one achieved a partial response, and seven maintained stable disease (109).

## 8 Conclusions

PPLG are rare neuroendocrine tumors with relevant clinical implications, characterized predominantly by the production of catecholamines, which can manifest in a spectrum of clinical symptoms.

Pheochromocytomas have the potential to be part of inherited syndromes such as MEN-2, VHL, and NF1, which implicate a variety of other pathologies and necessitate genetic screening of affected individuals and their family members. This genetic association requires a robust approach to diagnosis and treatment, integrating advanced imaging techniques, accurate laboratory testing, and detailed genetic analysis.

Treatment strategies for pheochromocytomas involve a multidisciplinary approach, including surgical intervention as the primary therapeutic option. Preoperative preparation with alpha-blockers and beta-blockers is crucial to mitigate the risks associated with catecholamine secretion during tumor manipulation. Non-surgical approaches, including chemotherapy and novel therapies such as tyrosine kinase inhibitors, play a role in the treatment of metastatic or inoperable cases, offering symptomatic relief and possible disease control.

Although PPGL are rare, their complex clinical presentations and potential genetic basis make them a significant challenge in endocrine and oncologic practice. Early diagnosis, a thorough understanding of the genetic landscape, and a comprehensive treatment strategy are critical to improving outcomes for patients with these potentially life-threatening conditions.
